# Human brain effects of DMT assessed via EEG-fMRI

**DOI:** 10.1073/pnas.2218949120

**Published:** 2023-03-20

**Authors:** Christopher Timmermann, Leor Roseman, Sharad Haridas, Fernando E. Rosas, Lisa Luan, Hannes Kettner, Jonny Martell, David Erritzoe, Enzo Tagliazucchi, Carla Pallavicini, Manesh Girn, Andrea Alamia, Robert Leech, David J. Nutt, Robin L. Carhart-Harris

**Affiliations:** ^a^Division of Psychiatry, Department of Brain Sciences, Centre for Psychedelic Research, Imperial College London, W12 0NN London, UK; ^b^Department of Informatics, University of Sussex, Brighton BN1 9RH, United Kingdom; ^c^Centre for Complexity Science, Imperial College London, London SW7 2AZ, United Kingdom; ^d^Center for Eudaimonia and Human Flourishing, University of Oxford, Oxford OX3 9BX, United Kingdom;; ^e^Departamento de Física, Latin American Brain Health Institute, Universidad Adolfo Ibanez, 3485 Santiago, Chile; ^f^Universidad de Buenos Aires and Instituto de Física de Buenos Aires, 1428 Buenos Aires, Argentina; ^g^Department of Neurology and Neurosurgery, Montreal Neurological Institute, McGill University, Montreal, QC H3A 2B4, Canada; ^h^CNRS Université de Toulouse, 31300 Toulouse, France; ^i^Department of Neuroimaging, Institute of Psychiatry, Psychology and Neuroscience, King’s College, London WC2R 2LS, UK; ^j^Psychedelics Division - Neuroscape, Department of Neurology, University of California, San Francisco, CA 94143

**Keywords:** psychedelics, serotonin, consciousness, dimethyltryptamine, ayahuasca

## Abstract

Psychedelics have attracted medical interest, but their effects on human brain function are incompletely understood. In a comprehensive, within-subjects, placebo-controlled design, we acquired multimodal neuroimaging [i.e., EEG-fMRI (electroencephalography-functional MRI)] data to assess the effects of intravenous (IV) N,N-Dimethyltryptamine (DMT) on brain function in 20 healthy volunteers. Simultaneous EEG-fMRI was acquired prior to, during, and after a bolus IV administration of 20 mg DMT, and, separately, placebo. At dosages consistent with the present study, DMT, a serotonin 2A receptor (5-HT2AR) agonist, induces a deeply immersive and radically altered state of consciousness. DMT is thus a useful research tool for probing the neural correlates of conscious experience. Here, fMRI results revealed robust increases in global functional connectivity (GFC), network disintegration and desegregation, and a compression of the principal cortical gradient under DMT. GFC × subjective intensity maps correlated with independent positron emission tomography (PET)-derived 5-HT2AR maps, and both overlapped with meta-analytical data implying human-specific psychological functions. Changes in major EEG-measured neurophysiological properties correlated with specific changes in various fMRI metrics, enriching our understanding of the neural basis of DMT’s effects. The present findings advance on previous work by confirming a predominant action of DMT—and likely other 5-HT2AR agonist psychedelics—on the brain’s transmodal association pole, i.e., the neurodevelopmentally and evolutionarily recent cortex that is associated with species-specific psychological advancements, and high expression of 5-HT2A receptors.

## Significance

This placebo-controlled multimodal [functional MRI-electroencephalography (fMRI-EEG)] human neuroimaging study offers the most comprehensive view of the acute brain action of psychedelics to date. It assessed N,N-Dimethyltryptamine (DMT), a psychedelic that generates immersive altered conscious experience with no diminishment of wakefulness. Global hyperconnectivity, collapsed hierarchical organization and reduced intranetwork integrity, was observed (fMRI) that correlated with decreased alpha power and increased entropy (EEG). Regions with the densest expression of serotonin 2A receptors as determined via independent positron emission tomography (PET) data, were most affected by DMT, and overlapped with regions related to evolved cognitive functions such as language and semantic processing. These results support the notion that psychedelics impact a principal axis of brain organization, and relatedly, the quality of human conscious experience.

N,N-Dimethyltryptamine (DMT) is a classic serotonergic psychedelic drug and useful consciousness probe. Plant-based DMT has likely been used for thousands of years in ceremonial and healing contexts (1, 2), and, like other psychedelics (3, 4), synthesized product is now being trialed as part of a drug-plus-psychotherapy combination for treating depression (5–7). DMT can induce an intense and immersive altered state of consciousness, characterized by vivid and complex imagery, and a sense of being transported to an alternative reality or dimension, without any diminishment in wakefulness (8). With sufficiently high doses, descriptions of encounters with sentient “beings” or “entities” are common (9–11). Such ontologically shocking experiences have been found to correlate with subsequent changes in metaphysical beliefs (12), and mental health (12), and formal comparisons have been made between the phenomenology of DMT experiences and near-death experiences (9) and the dream state (13).

Clinical trials of psychedelic therapy for the treatment of a variety of mental health conditions have yielded consistently promising safety and efficacy findings (3, 14). The signature psychological effects of the so-called classic psychedelics are initiated via agonism at the serotonin 2A receptor (5-HT2AR) (2, 15). A wealth of convergent evidence suggests that 5-HT2A receptor agonism is the trigger event in psychedelics’ therapeutic action, likely initiating processes of cortical plasticity that are exploited via adjunctive psychological support (16–18).

Most human research with pure DMT has involved an intravenous mode of administration. Given this way, the drug has a rapid action—peaking at ~3 min and subsiding at ~15 min; ideal for examining rapid changes in brain function associated with a rapid transition into, and back from, a highly altered quality of waking consciousness. Accordingly, human functional neuroimaging with DMT offers a unique scientific opportunity for advancing our understanding of the neurobiology of conscious states.

Results from previous functional MRI (fMRI) studies assessing the brain effects of psychedelics have revealed decreased within-network functional connectivity (FC) or “integrity” (19, 20), increased between-network FC or decreased “segregation” (19, 21), a larger repertoire of brain connectivity profiles (22–24), a globally hyperconnected brain state (25), freer transitions between brain states (24, 26), and a reduction of its principal sensory-association cortex gradient (27). Importantly, these studies broadly converge on the action of psychedelics on the transmodal association cortex pole (or “TOP”) of the human brain. The TOP sits at the upper end of a hierarchical gradient of cortical organization, while unimodal sensory areas sit at the lower end. The TOP is linked to abstract semantic representations, longer temporal windows of information processing, and is relatively more detached from sensory input, while also appearing later in primate cortical expansion and development (28, 29). These findings suggest that the subjective effects of psychedelics depend on the dysregulation of the association cortices. Evidence from neuroimaging studies also suggests that this cortical dysregulation may result in the disinhibition of “lower” or evolutionarily and developmentally “earlier” systems such as the so-called limbic system (30).

Until the present study, only indirect correlations have been possible between electrophysiological and fMRI functional brain activity recordings done under the effects of a psychedelic (19); thus, the present study offers an opportunity to simultaneously acquire and then correlate such data, while at the same time leveraging DMT’s short-acting effects. Acquiring electroencephalography (EEG) and fMRI in parallel also enables us to see true neuronal activity (i.e., through the EEG data) rather than having to infer it through a hemodynamic signal [i.e., the fMRI bloodoxygen level–dependent (BOLD) signal] that could be confounded by a direct vascular action of the drug (31). Furthermore, the high temporal resolution of EEG provides reliable means to relate the rhythmic and entropy-related effects induced by a psychedelic (32–34) with simultaneous changes in brain connectivity captured with fMRI. The inclusion of EEG therefore gives us confidence about the neuronal action of the psychedelic.

In this study, we advance on previous work examining the neural correlates of the psychedelic state (19, 20) by combining fMRI and electroencephalography (EEG) in a simultaneous recording paradigm in a resting-state condition. Intravenous DMT (versus placebo) was administered to healthy volunteers during eyes-closed, resting-state conditions. This approach offers an important advancement because it enables the direct observation of changes in neuronal activity (EEG) in parallel with indirect changes seen through the fMRI blood oxygen level–dependent (BOLD) signal. Further, the EEG-fMRI combination overcomes each modality’s bounds on temporal or spatial resolution—enabling the collection of the most comprehensive information possible on brain activity via noninvasive contemporary human functional neuroimaging.

## Results

### fMRI: Static Resting-State Functional Connectivity (sRSFC).

**Fig. 1.**
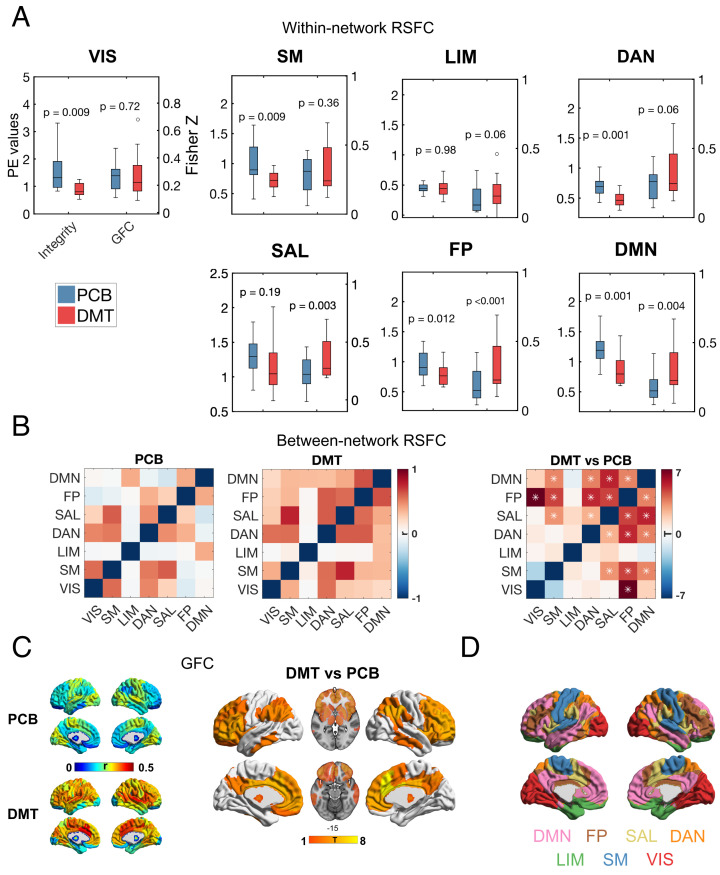
Reduced RSN integrity and segregation and increased GFC with DMT. (*A*) Analysis of within-network sRSFC or integrity (parameter estimates and Fisher Z values) for DMT (red) versus placebo (blue) shows significant reductions in integrity for 5 of 7 networks, and increases in global functional connectivity (GFC) in 3 of 7 networks (FDR correction, *P* < 0.05). (*B*) Decreased between-network segregation was especially pronounced for the FP/DMN/SAL or TOP networks and other networks (*P < 0.05, FDR corrected). (*C*) Increases in GFC were especially pronounced for regions associated with the TOP of the human brain’s principal gradient (*P* < 0.05, FDR corrected). See *SI Appendix*, Figs. S2 and S3 for complementary analysis without motion confounds and *SI Appendix*, Fig. S5 for analysis using global signal regression. (*D*) Networks used for analyses (sRSFC = static resting-state functional connectivity; networks; VIS = visual; SM = somatomotor; DAN = dorsal attentional; SAL = ventral attentional/salience; LIM = limbic; FP = frontoparietal; DMN = default mode; TOP = transmodal association pole).

Twenty participants (mean age = 33.5 y, SD = 7.9, 7 females) were given a high dose (20 mg) of DMT fumarate and placebo in a within-subjects, counterbalanced, pseudo-randomized design. Simultaneous fMRI and EEG recordings took place from 8 min before until 20 min after the DMT/placebo injection (see *SI Appendix*, Fig. S1 for subjective effects). To determine the effects of DMT on brain connectivity, using the fMRI BOLD signal, we analyzed resting state functional connectivity (RSFC) averaged over an 8-min period after the injection of DMT/placebo, coinciding with the time of peak subjective intensity. To differentiate this time-averaged analytical approach from more dynamic metrics, we name it “static” RSFC or “sRSFC.” For within-network RSFC or integrity and between-network RSFC or segregation, we used independent component analysis (ICA) to derive a set of canonical resting-state networks (RSNs), followed by the “dual regression” approach to examine RSFC changes (35). To complement these analyses, we computed global functional connectivity (GFC), which indexes the average RSFC (i.e., correlation) for a given region with all other regions in the brain (see *Materials and Methods* for details). Compared with placebo, DMT significantly decreased the within-network integrity of all canonical RSNs (*P* < 0.05, FDR corrected), with the exception of the salience (SAL) and limbic (LIM) networks. Significant increases in GFC were found in the SAL, frontoparietal (FP), and default-mode networks (DMN; *P* < 0.05, FDR corrected) (Fig. 1*A*), overlapping the transmodal association cortex pole (or TOP) of the human brain’s principal RSFC gradient (36). Additionally, DMT was found to decrease between-network segregation, especially for frontoparietal, salience, and default-mode networks (Fig. 1*B*) (*P* < 0.05, FDR corrected), again implicating the transmodal pole.

Analyses of GFC at the level of individual brain regions [following Schaefer et al. (37) parcellation] confirmed the hyperconnectivity of TOP regions (*P* < 0.05, FDR corrected) (Fig. 1*C*). Finally, whole-brain GFC (i.e., average GFC across all regions) significantly increased in DMT compared with placebo [t(15) = 3.11, *P* = 0.007, 95% CI = [0.028 0.15]].

Due to potential head-motion confounds, we replicated static RSFC analysis employing two different subsamples of participants that presented no significant motion confounds (*Materials and Methods*). Consistent findings with those reported in the main results section of this paper were found (*SI Appendix*, Figs. S2 and S3), and head motion was not significantly different between DMT and placebo in both of these subsamples (*SI Appendix*, Fig. S4). Furthermore, after applying global signal regression (GSR), we found consistent findings (*SI Appendix*, Fig. S5). Across all analyses, DMN and dorsal attentional network (DAN) disintegration was observed; increases in GFC were ubiquitous in DMN and FP networks; and regional GFC increases were found in the medial prefrontal cortex, dorsolateral prefrontal cortex, insula, and temporoparietal junction.

### fMRI: Dynamic Resting-State Functional Connectivity (dRSFC).

**Fig. 2.**
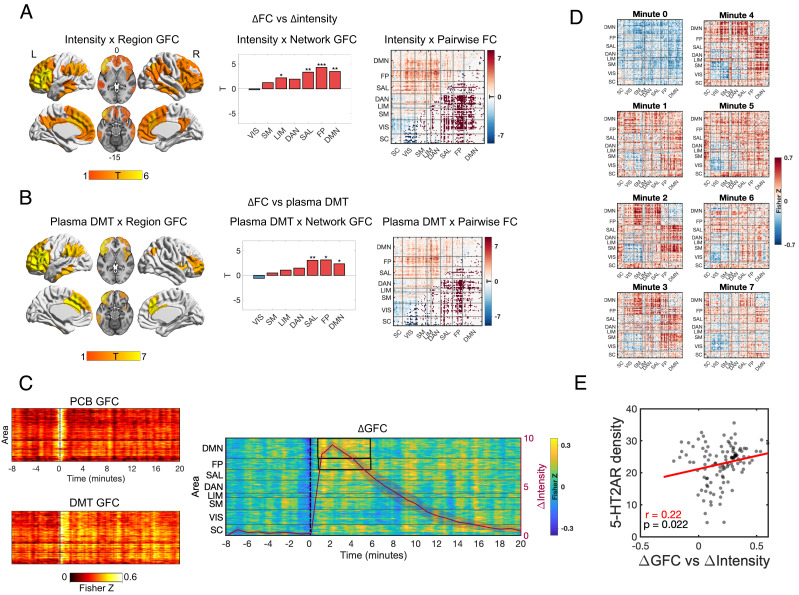
Dynamics changes in brain RSFC under DMT. (*A*, *Left*) Areas showing a significant association between real-time intensity ratings and dynamic global functional connectivity (GFC) for DMT minus placebo (*P* < 0.05, FDR corrected). (*A*, *Middle*) Dynamic effects of DMT versus placebo on network GFC, showing significant increases in GFC for DMN/FP/SAL networks—all associated with the TOP of the brain’s principal functional gradient, as well as the LIM network (*P < 0.05, **P < 0.01, ***P < 0.001; FDR corrected). (*A*, *Right*) Pairwise functional connectivity (FC) matrix representing the association between intensity ratings and dynamic functional connectivity (significant links are highlighted in the lower diagonal; FDR corrected). (*B*) Areas showing a significant association between DMT plasma levels and regional GFC, network GFC, and pairwise FC (FDR corrected). (*C*, *Left*) Regional GFC across time for placebo, DMT, and (*C*, *Right*) DMT minus placebo, overlayed with reported average intensity ratings (±SEM). Black boxes highlight epochs where changes in network GFC are statistically significant (cluster corrected, *P* < 0.05). (*D*) Average (DMT—placebo) pairwise FC matrices for the first seven minutes following DMT/placebo administration. (*E*) A significant association was found between 5-HT2AR density maps and dynamic GFC (representing beta values of the relationship between GFC and intensity ratings). (Networks: VIS = visual; SM = somatomotor; DAN = dorsal attentional; SAL = ventral attentional/salience; LIM = limbic; FP = frontoparietal; DMN = default mode; SC = subcortical regions; TOP = transmodal association pole).

To better examine the dynamic effects of DMT on brain function, mappings were made between real-time verbally reported subjective intensity ratings (*Materials and Methods*) and dynamic functional connectivity (dRSFC) measured via a sliding window approach. Both GFC (per region and per network) and pairwise functional connectivity metrics were calculated (per link; see *Materials and Methods* for details). Significant positive correlations between DMN/FP/SAL/LIM GFC and real-time intensity ratings were found (*P* < 0.05, FDR corrected), i.e., increased DMN/FP/SAL/LIM GFC correlated with greater intensity. Widespread significant positive associations were evident between intensity ratings and pairwise connectivity across the brain; however, a negative association was found between intensity and pairwise connectivity of visual (VIS)-somatomotor (SM)/subcortical regions (*P* < 0.05, FDR corrected) (Fig. 2*A*). Supplementing these results, correlations were seen between selective dRSFC changes and average plasma concentration levels of DMT (Fig. 2*B*).

Regarding specific dynamics, increased DMN and FP GFC were greatest during the initial minutes post DMT injection compared to placebo (~1 to 6 min) (*P* < 0.05, cluster corrected) (Fig. 2*C*). Pairwise connectivity analyses revealed increases in GFC across most brain areas during the first minute, while segregation between lower-order unimodal sensory and motor areas became more apparent from minute two onward (Fig. 2*D*).

An averaged binding potential or “density” map for the 5-HT2A receptor was derived from previous positron emission tomography (PET) imaging work in humans (38). Relationships were examined between this in vivo map and dynamic GFC results from the present study. Results evidenced a significant positive relationship between 5-HT2A receptor density and a GFC regression model with intensity ratings entered as an explanatory variable. This result implies a (logical) causal effect of DMT-induced 5-HT2AR stimulation on increased GFC and its relationship with the subjective intensity of the psychedelic state (Fig. 2*E*).

Finally, exploiting the NeuroSynth tool—a database of coordinates of brain activations associated with functional terms (https://neurosynth.org/)—we examined associations between both GFC and 5-HT2A receptor density maps, and function. The first 30 (nonanatomical) functional terms that emerged as being most closely related to the GFC and 5-HT2A receptor maps are shown in *SI Appendix*, Fig. S6 (see *SI Appendix*, Fig. S7 for exploratory correlations between psychometric measures and imaging metrics). Both maps highlighted high-order cognitive and linguistic functions in keeping with a fundamental impact of DMT on the TOP of the human brain’s principal gradient.

### fMRI: Principal Cortical Gradient.

**Fig. 3.**
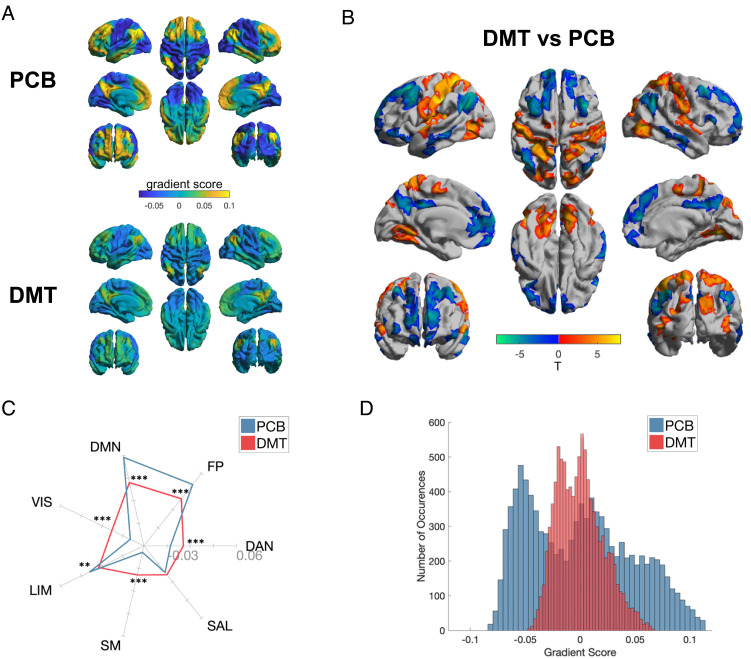
Compression of the principal gradient under DMT. (*A*) Mean principal gradient for the placebo (*Top*) and DMT (*Bottom*) conditions, representing the principal axis from unimodal to transmodal cortex. (*B*) DMT > Placebo between-group vertex-wise (*Top*) and (*C*) network-wise (*Bottom*) contrasts. Network-wise radial plot displays the mean intranetwork principal gradient score for each network for DMT and placebo (*P < 0.05, **P < 0.01, ***P < 0.001; FDR corrected). (*D*) Histogram showing the distribution of principal gradient values for placebo and DMT conditions for each brain area. (Networks: VIS = visual; SM = somatomotor; DAN = dorsal attentional; SAL = ventral attentional/salience; LIM = limbic; FP = frontoparietal; DMN = default mode).

Given the appearance of a fundamental action of DMT on global brain organization, we proceeded to directly examine changes in the brain’s principal sensorimotor to association cortex gradient. Results revealed a whole-brain action of DMT and, more specifically, a compression of the brain’s principal or fundamental sensory-association axis or gradient. To execute this analysis, we applied gradient-mapping analyses following previous work (27, 36). As described in *Materials and Methods*, this approach involves the application of a nonlinear dimensionality reduction algorithm—“diffusion map embedding.” Inputs to this algorithm are subject-wise interregional FC similarity matrices, wherein each value represents the similarity in whole-brain FC between two given regions. The outputs of this algorithm are “gradients” of continuous variation across the cortex, each representing a particular axis of covariance in functional connectivity (dis)similarity. Notably, the principal axis revealed by this approach represents a hierarchical axis spanning from a low-level sensorimotor pole to a high-level transmodal association cortex pole (which we refer to here as TOP). A large number of properties of human brain function, anatomy, development, and evolution appear to match this sensorimotor to association cortex gradient (28). In the present results, high-order TOP regions displayed reduced gradient scores (consistent with a movement toward zero), while low-level unimodal regions displayed increased gradient scores (consistent with a movement toward zero)—accounting for an overall axis compression (Fig. 3 *A* and *B*). Network-based analyses further explicated this compression of the cortical hierarchy, with increased gradient scores within unimodal sensory networks (e.g., somatomotor; SM, and visual; VIS), as well as the DAN, and decreased scores within the FP, DMN, and LIM (Fig. 3*C*). These analyses imply a reduction in unimodal–transmodal differentiation and macroscale hierarchical organization in the brain (Fig. 3*D*).

### EEG: Spectral Power, Signal Diversity, and Cortical Traveling Waves.

**Fig. 4.**
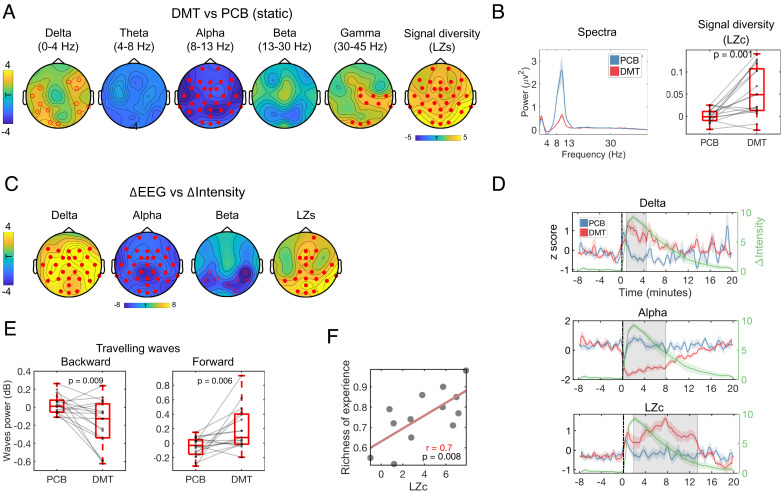
Effects of DMT on EEG spectral power and signal diversity. (*A*) DMT-induced widespread decreases in alpha power and increases in signal diversity (estimated via Lempel–Ziv, [LZ]) plus delta and gamma power. (*B*) Whole-brain power spectra and signal diversity (LZc) showed consistent between-condition differences. (*C*) Minute-by-minute intensity ratings correlated positively with increases in delta power and LZs and negatively with global alpha and posterior beta power changes (•*P* < 0.01, º*P* < 0.05, cluster corrected). (*D*) Temporally resolved effects of DMT on delta and alpha power, plus LZc (shaded areas correspond to epochs of between-condition statistical difference, *P* < 0.05, cluster corrected). The green trace reflects average subjective intensity ratings over time. (*E*) Significantly backward wave (BW) power and increased forward wave (FW) power was observed in the DMT versus placebo contrast (each condition was baseline corrected). (*F*) Correlation between LZc and “richness of the experience.” LZs = Lempel–Ziv per channel; LZc = Lempel–Ziv averaged across channels.

The effects of DMT on EEG measured brain activity included static (average in the 8 min postinjection), dynamic (intensity ratings vs. EEG measures), and temporally resolved (for detecting periods of significant differences) analyses. In all the three cases, data recorded under DMT were contrasted with data recorded under placebo (see *Materials and Methods* for details). Consistent with previous DMT study findings (32, 39), static analyses revealed widespread decreases in alpha power under DMT (*P* < 0.01, cluster corrected), and increases in both delta (*P* < 0.05, cluster corrected) and gamma bands (*P* < 0.01, cluster corrected). Additionally, widespread increases in signal diversity, determined via Lempel–Ziv complexity (denoted hereafter as “LZc” for the global average and “LZs” for single channels), were seen for DMT versus placebo (*P* < 0.01, cluster corrected) (Fig. 4 *A* and *B*). Furthermore, dynamic analyses (intensity vs EEG measures) corroborated these findings, and further revealed positive correlations between real-time intensity ratings and both delta power and LZc changes, i.e., more intense experiences were associated with greater increases in delta power and LZc. Negative correlations between global alpha and posterior beta power changes and subjective intensity were also seen, with more intense experiences linking to greater decreases in alpha and beta power (*P* < 0.05, cluster corrected (Fig. 4*C*).

Focusing on specific epochs of statistical significance, alpha power reductions (~0 to 8 min, *P* = 0.001, cluster corrected) and LZc increases (~2 to 14 min, *P* = 0.001, cluster corrected) separated from placebo for a greater proportion of the overall experience than did delta power increases—which only separated during the onset phase (~0 to 4 min, *P* = 0.006, cluster corrected) (Fig. 4*D*). Additionally, marginally significant reductions in beta power (~0 to 1 min, *P* = 0.04, cluster corrected) were observed during the first minute post-DMT administration.

Additional analyses found significantly reduced backward traveling wave power under DMT and increased forward traveling wave power, consistent with previous results (40) (Fig. 4*E*). Finally, consistent with a strong prior hypothesis (41), signal diversity (averaged over the 8 min postinjection) was correlated with ratings of the visual analog scale item “How rich was your conscious experience” (r = 0.7, *P* = 0.008; Fig. 4*F*).

### Parallel Changes in EEG & fMRI.

**Fig. 5.**
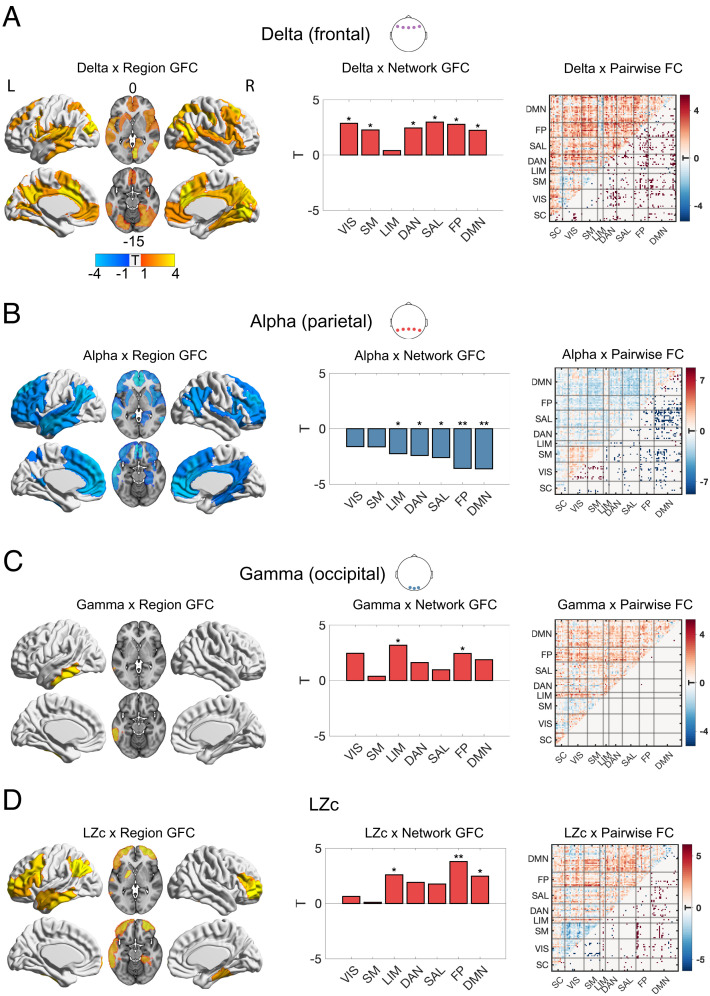
EEG changes in relation to fMRI RSFC changes. Displayed in *A*–*D* are associations between EEG measures and global functional connectivity (GFC) per region (left brain surfaces; significant regions displayed; *P* < 0.05, FDR corrected), network GFC (middle bar plots; significant *P* < 0.05, FDR corrected), and pairwise functional connectivity (FC) (right correlation matrices with significant links displayed in the lower quadrants; *P* < 0.05, FDR corrected). (*A*) Frontal delta power was positively associated with widespread GFC and distributed connections. (*B*) Parietal alpha power was negatively associated with GFC in high-level and attentional networks, as well as the limbic network. (*C*) Occipital gamma power was positively associated with increases in GFC at frontoparietal and limbic networks. (*D*) Signal diversity (LZc) was associated with increases in GFC at high-level and limbic networks (*P < 0.05, **P < 0.01, ***P < 0.001; FDR corrected). (Networks: VIS = visual; SM = somatomotor; DAN = dorsal attentional; SAL = ventral attentional/salience; LIM = limbic; FP = frontoparietal; DMN = default mode; SC = subcortical regions)

Exploiting this study’s unique simultaneous recording of EEG and fMRI data, the time course of delta, alpha, beta, and gamma band activity, as well as global signal diversity (LZc), was analyzed in conjunction with fMRI measures of functional connectivity, with a focus on GFC and pairwise FC in the whole brain. Fig. 5 displays correlations using data from the electrodes and EEG metrics that displayed the most pronounced effects (*Materials and Methods*).

Regression models incorporating key EEG metrics as explanatory variables of changes in key fMRI metrics, yielded consistent findings implicating changes in the brain’s principal sensory-association gradient. Specifically, average delta power measured at frontal electrodes was found to be positively associated with a pattern of widespread GFC incorporating frontal, temporal, parietal, motor, and visual association cortices, and this finding was replicated in the pairwise FC results (*P* < 0.05, FDR corrected) (Fig. 5*A*). Average alpha power changes (decreases) at parietal electrodes were negatively related to GFC changes (increases) in TOP networks and regions. For low-level regions, a more complex relationship emerged, as alpha power reductions were associated with decreased coupling between visual and somatomotor regions, and increased coupling between these sensory and TOP regions (*P* < 0.05, FDR corrected; see pairwise FC correlation matrix in Fig. 5*B*). Gamma power changes (increases) in occipital electrodes were associated with changes in GFC (increases) in FP regions and the LIM network (*P* < 0.05, FDR corrected) (Fig. 5*C*). Signal diversity (LZc) changes (increases) were associated with changes in GFC (increases) in TOP networks, as well as the LIM network (*P* < 0.05, FDR corrected) (Fig. 5*D*). In brief, these relationships mirror the modality independent changes in both EEG and fMRI measured activity (Figs. 1–4) but advance on these in an important and meaningful way, by demonstrating that the directionality of change within each metric significantly interrelates between modalities. The complement of simultaneous EEG alongside fMRI also enables an appreciation of neuronal changes (EEG) paralleling network effects viewed through fMRI’s BOLD signal. Furthermore, we leveraged DMT’s short-acting effects to exploit within-subject trajectories in both modalities and find meaningful relationships between EEG and fMRI, which are not evident when employing an averaging approach for between-subject correlations (*SI Appendix*, Figs. S8 and S9).

## Discussion

Utilizing two complementary imaging modalities performed in tandem (EEG-fMRI), the present study offers an advanced view of the acute action of a psychedelic on the human brain. Its results provide an update on the current thinking regarding the neural correlates of the psychedelic state, consolidating some relatively well-established properties—such as network disintegration and desegregation, decreased alpha power and increased spontaneous signal complexity or entropy, as well as relationships between metrics (19–21, 34, 42)—but also strengthening confidence in some more recent observations—such as altered traveling waves (40) and increased delta (32) and gamma power (39), and global functional connectivity (GFC) (25).

Perhaps, the most enlightening aspect of the present results, however, is how they converge on a *global* brain action of psychedelics, implicating dysregulation of activity in the brain’s transmodal association cortex pole (TOP). While decreased communication between low-level sensorimotor modules at the lower end of the cortex’s principal organizational gradient was also seen, dynamic and time-resolved analyses suggested that these effects may occur subsequent in time to a primary action at the brain’s TOP.

Psychedelics are particularly useful research tools for studying the neurobiology of consciousness. Their ability to shift its quality in a fundamental and often enduringly transformative way, while preserving wakefulness, is arguably unparalleled in pharmacology. We believe the present results, and particularly those pertaining to whole-brain organization, such as global FC, pairwise FC, and the principal FC gradient, now point toward a signature global brain action of psychedelics that is robust, reliable, unique, and revealing, not just of “psychedelic consciousness” but of conscious experience more broadly.

A wealth and diversity of convergent data is now highlighting the human cortex’s principal functional gradient (36), spanning from a lower-order sensorimotor cortex pole to a higher-order transmodal association cortex pole, as a defining organizational dimension of the human brain (29). Covering all the relevant properties that match this principal gradient, dimension, or axis, is beyond the scope of the present paper, but some important examples include: primate to human cortical expansion; ontogenetic cortical expansion; various aspects of developmental maturation – including prolonged plasticity periods, myelination degree, metabolism and blood flow, excitatory neurotransmission (29); serotonin 2A receptor expression (38); the degree of abstraction (43) and temporal duration (44) of information processing [see Sydnor et al. (28) for a review]; and traveling waves linked to infraslow cycles of arousal (45).

The present study is not the first to apply whole-brain metrics to psychedelic neuroimaging data and show their relationship to signature aspects of the psychedelic experience (25), and neither is it the first to propose that a profound whole-brain action may differentiate classic psychedelics from analogous compounds such as MDMA (21); however, it addresses the global brain action of a psychedelic in a comprehensive multivariate and multimodal way, twinned with an especially immersive psychedelic experience.

The picture that emerges is one of a drug-induced collapse of the defining principal gradient of the brain, such that its transmodal association cortex pole becomes less segregated from—or more integrated with—the rest of the cortex. The TOP is, per definition, transmodal in nature and evolutionarily “new” or recent, whereas the lower-order cortex is unimodal and older (28). That in vivo PET maps of 5-HT2A receptor distribution overlap with the principal functional gradient, as well as the global FC effects of psychedelics (Fig. 2*E*), implies that psychedelic-induced increases in 5-HT2AR signaling are causal of the relevant functional effects; an assumption backed by computational modeling (46).

In the present study, we found that dysregulation of brain activity (e.g., indexed via reductions of alpha power, as well as by increases in gamma power and signal diversity) was associated with increased global functional connectivity at the brain’s TOP. These findings are indicative of a transition to a more entropic mode of brain functioning under psychedelics which may account for their distinct phenomenology (41, 47). We also found increases in (frontal) delta power following DMT; these increases were correlated with a state of increased global connectivity (fMRI), consistent with the notion that increased delta may serve as a marker for a significant alteration of consciousness, rather than one exclusively linked to reduced conscious level—as traditionally thought (48). Simultaneously, we found that alpha power reductions, gamma power increases, and signal entropy enhancements were related to higher GFC in the limbic network (Fig. 5 *B*–*D*), consistent with the hypothesis that the emergence of psychedelic phenomena may involve limbic disinhibition, with implications for memory and emotion processing (30). Broadly, these findings converge on the notion that DMT-induced-5-HT2AR agonism dysregulates high-level cortical activity and limbic activity.

There are plentiful theories on the uniqueness of the human brain, mind, and behavior—e.g., see Sydnor et al. (28) for a review. Flexible and capacious information processing (28, 49)—particularly in semantic and linguistic domains – is one candidate (28). It may be telling therefore that both the *GFC × intensity* and 5-HT2A receptor spatial maps overlapped with NeuroSynth functional terms related to cognitive faculties that have evolved greatly in our species, e.g., “language” and “semantic.”

Recent findings suggest that 5-HT2AR signaling plays a direct, receptor and species selective role in early phases of cortical expansion, e.g., increasing the proliferation of basal progenitor cells (50). This raises the possibility that the key target of psychedelics—the 5-HT2A receptor—has played some causal role in the expansion of the human cortex. The abundance of cortical neurons, particularly in the TOP of the cortex [20-fold increase from macaque to human (50)], is a defining feature of the human brain. The densest expression of 5-HT2A receptors can be found in the TOP of the cortex, and, as the present study has shown, this is also where psychedelics have their initial and most robust effects. Increased synaptic growth via 5-HT2AR agonism (16, 17, 51) implicates the receptor in ontogenetic brain development and learning (52).

Indeed, there is now a wealth of evidence linking 5-HT2AR signaling with the induction of various aspects of neural and behavioral plasticity (16, 17, 51), but whether such effects are functionally advantageous or impairing is a complex question dependent on several factors. Relatedly, the acute and longer-term psychological effects of psychedelics are thought to be highly context sensitive and dependent (53), explaining why such emphasis is placed on “set and setting” in psychedelic therapy (54, 55). It is important to note that 5-HT2AR is also densely expressed in the primary visual cortex (*SI Appendix*, Fig. S6), and DMT is known for inducing vivid visual imagery. Further studies are required to examine the specific DMT-induced increased connectivity we found between the TOP of the cortex and visual areas (Fig. 2 *A* and *B*), which may help explain the visual quality of the DMT experience (19).

The question of what is specific to the action of DMT versus other classic psychedelics is difficult to answer from the present study alone, with its exclusive focus on DMT and lack of an active drug control condition. Most of this study’s findings are consistent with previous observations on psychedelic neuroimaging data, albeit with a higher fidelity and multilevel depth—afforded by the study’s design and the subjective potency of 20 mg of I.V. DMT. Increased delta power may be DMT specific, but more research is needed to test this (e.g., see refs. 32, 39, and 48).

With regard to other drugs and states, the pattern and quality of effects seen here with DMT and EEG-fMRI are quite distinct from those seen previously with a serotonin reuptake inhibitor (56), stimulant (57), sedative (58), dissociative (59) [although see Forsyth et al. (60)], as well as MDMA (21). It is intriguing to speculate that increased global functional connectivity in high-level regions and networks may be a somewhat exclusive property of the action of classic psychedelics. However, a broadly similar profile of brain function (i.e., dysregulation of activity in high-level cortex and compression of the brain’s hierarchical organization) has been seen in experienced meditators meditating (61)—as well as in schizophrenia, (62) and infancy (63).

Importantly, validation checks were performed to assess the influence of motion confounds, as well as global signal regression (GSR). Relevant results can be found in the *SI Appendix*, Figs. S2–S5, S8, and S10. Previous psychedelic GFC results have been published (64, 65) that have shown different patterns of change than those observed here with DMT and previously with LSD and psilocybin (25)—although a recent independent study with psilocybin reported broadly convergent results with our own (66). One potentially important difference in these studies’ analytical approaches is whether or not they performed GSR. Due to assumptions that the global signal contains functionally meaningful neuronal information (67) that may be sensitive to modulation via a potent intervention, and that other preprocessing procedures exist to address global “noise,” we chose not to perform GSR in our main analyses. Instead, our thorough preprocessing included a draining veins regressor that other work has shown covaries with the global signal—yet is more clearly nonneuronal and therefore the more logical nuisance regressor. Regardless, we performed and reported the results of GSR in the *SI Appendix*, Fig. S5. Briefly, after conducting GSR, results remained largely consistent with those reported here in the main results section of this study and were inconsistent with GFC results previously published with LSD and psilocybin when GSR was performed (64, 65). Data and analytical pipeline sharing is currently underway in an effort to resolve between-team results discrepancies.

DMT increases peripheral markers of arousal, and some of our imaging findings are consistent with increases in arousal, such as decreased alpha power, DMN integrity, and increased LZ. However, the breadth and magnitude of the brain changes observed here with DMT surpass what one would expect from mere arousal. Importantly, in validation analyses, we did not find compelling associations between drowsiness ratings and between-condition effects on various imaging metrics (*SI Appendix*, Table S1). Furthermore, findings of increased delta power and associated hyperconnectivity are inconsistent with either increases in arousal or decreases in drowsiness (48). The current results with DMT are consistent with previous findings of altered brain function on LSD and psilocybin, and these previous studies did not consistently observe increases in markers of arousal (19–21, 25). Nevertheless, further work is needed to parse specific psychedelic effects in the brain from mere increases in arousal or decreases in drowsiness.

It is important to note that fMRI connectivity metrics are highly sensitive to head motion and while we did find that head motion was significantly larger under DMT versus placebo, analysis of subsamples comprised participants with comparable head motion across conditions revealed consistent findings with those reported in the main *Results* section (*SI Appendix*, Figs. S2 and S3). Future research studies should endeavor to collect larger datasets with more participants, ideally with equivalent head motion between conditions.

While this study found a significant relationship between changes in global functional connectivity induced by DMT and ratings of intense subjective effects, few correlations were found between imaging metrics and specific subjective effects (e.g., visual imagery, “entity” encounters, feelings of immersion; *SI Appendix*, Fig. S7). Future studies—combining neuroimaging with time-resolved measures of subjective experience and/or experience-sampling plus extensive within-subject data collection—could leverage improvements in data volume and quality for more nuanced “neurophenomenological” analyses (68).

Finally, a minor component of the present study’s results was its direct testing of the so-called “entropic brain hypothesis,” which postulates that psychedelics elevate the entropy of spontaneous brain activity in parallel with increases in the “richness” of conscious experience (41, 47)—where richness is defined as depth of content. Here, we found clear support for this hypothesis via significant correlations between the self-rated richness of conscious experience and LZc increases (Fig. 4*F*)—a useful marker of the entropy or complexity of spontaneous brain activity (41).

In conclusion, the present multimodal, multivariate EEG-fMRI study with DMT revealed a robust dysregulating effect on activity in the brain’s TOP, where 5-HT2A receptors (the main target of psychedelics) are most densely expressed. Observations of increased communication between the TOP of the cortex and the rest of the brain, could be interpreted as evidence of expanded information processing and a hyperassociative style of cognition. It is intriguing to speculate whether the magnitude of these global brain changes relates to increased *plasticity* (i.e., the property of being easily shaped or molded) in both a neuronal and behavioral sense. This study’s results consolidate the view that psychedelics target and dysregulate developmentally and evolutionary recent cortex. Moreover, they imply that the normal functioning of this high-level cortex may be necessary for the preservation of human-specific psychological faculties, but not wakeful conscious experience itself.

## Materials and Methods

### Participants and Experimental Procedures.

This was a single-blind, placebo-controlled, counter-balanced design. An initial visit at the Imperial College Research Facility was focused on assessing physical and mental health to ensure suitability. Exclusion criteria included: <18 y old at the moment of participation, MR contraindications, absence of experience with a psychedelic, an adverse reaction to a psychedelic, history of psychiatric or physical illness rendering unsuitable for participation (i.e., diabetes, epilepsy, or heart disease), family history of psychotic disorder, or excessive use of alcohol or drugs of abuse. All participants provided written informed consent for participation in the study. This study was approved by the National Research Ethics Committee London—Brent and the Health Research Authority and was conducted under the guidelines of the revised Declaration of Helsinki (2000), the International Committee on Harmonization Good Clinical Practices guidelines, and the National Health Service Research Governance Framework. Imperial College London sponsored the research, which was conducted under a Home Office license for research with Schedule 1 drugs.

Volunteers participated in two testing days at the Imperial College Clinical Imaging Facility, separated by two weeks. On each testing day, participants arrived and were tested for drugs of abuse and were involved in two separate scanning sessions. In this initial session (task free), they received intravenous (IV) administration of either placebo (10 mL of sterile saline) or 20 mg DMT (in fumarate form dissolved in 10 mL of sterile saline)—injected over 30 s, and then flushed with 10 mL of saline over 15 s—in a counter-balanced order (half of the participants received placebo and the other half received DMT). This first session always consisted of continuous resting-state scans which lasted 28 min with DMT/placebo administered at the end of 8th min and scanning was over 20 min after injection. Participants laid in the scanner with their eyes closed (an eye mask was used to prevent eyes opening), while EEG activity was recorded. Following the scanning procedure, participants were interviewed and completed questionnaires designed to assess the subjective effects experienced during the scan [Visual Analog Scales and validated scales: 11 Dimensions Altered States of Consciousness Questionnaire—ASC-11D (69) and the Mystical Experience Questionnaire—MEQ-30 (70)]. A second session then followed with the same procedure as the initial session (including scanning conditions), except on this occasion participants were (audio) cued to verbally rate the subjective intensity of drug effects every minute in real time while in the scanner. These ratings were then used for analysis in the present report (71).

This article reports the results concerning the resting-state scans in which no intensity ratings were asked, while using intensity ratings collected in other (nonanalyzed) scan runs as covariates for dynamic fMRI and EEG analysis. In total, 20 participants completed all study visits (7 female, mean age = 33.5 y, SD = 7.9) (71).

### fMRI and EEG Acquisition.

Images were acquired in a 3T MR scanner (Siemens Magnetom Verio syngo MR B17) using a 12-channel head coil for compatibility with EEG acquisition. Functional imaging was performed using a T2*-weighted BOLD-sensitive gradient echo planar imaging sequence [repetition time (TR) = 2000ms, echo time (TE) = 30 ms, acquisition time (TA) = 28.06 mins, flip angle (FA) = 80°, voxel size = 3.0 × 3.0 × 3.0mm3, 35 slices, interslice distance = 0 mm]. Whole-brain T1-weighted structural images were also acquired (71).

EEG was recorded inside the MRI environment during image acquisition. EEG data were recorded at 31 scalp sites following the 10 to 20 convention with an MR-compatible BrainAmp MR amplifier (BrainProducts, Munich, Germany) and an MR-compatible cap (BrainCap MR; BrainProducts GmbH, Munich, Germany). This system referenced all electrodes to FCz and AFz served as ground electrode. We additionally recorded with two additional ECG channels to improve heart rate acquisition for artifact minimization during EEG preprocessing, and all impedances were kept below 20kΩ. EEG was sampled at 5 kHz and with a hardware 250 Hz low-pass filter. EEG-MR clock synchronization was ensured using the Brain Products SyncBox hardware. Additional recordings of 5 min eyes-closed resting-state were performed outside of the scanner before DMT/placebo was administered in order to determine the profile of EEG activity in the time and frequency domain and ensure that artifact minimization procedures achieved a similar profile (71).

### fMRI Preprocessing.

The same preprocessing pipeline as used in previous work with LSD (19) was used here. Four out of 20 participants were discarded from group analyses due to excessive head movement during the 8-min post DMT timeperiod shown in Fig. 1 [>20% of scrubbed volumes with a scrubbing threshold of frame-wise displacement (FD) of 0.4 (72)]. Three further participants were removed for dynamic analysis after reaching a 20% threshold for total amount of scrubbed volumes for the full 28 min of scanning. Preprocessing steps consisted of 1) despiking [3dDespike, Analysis of Functional NeuroImages (AFNI) (73)]; 2) slice time correction [3dTshift, AFNI (73)]; 3) motion correction [3dvolreg, AFNI (73)] by registering each volume to the most similar volume, in the least squares sense, to all others (in-house code); 4) brain extraction [BET, FSL (74)]; 5) rigid body registration to anatomical scans; 6) nonlinear registration to 2mm MNI brain [Symmetric Normalization, Advanced Normalization Tools (ANTS) (75)]; 7) scrubbing—using an FD threshold of 0.4 and scrubbed volumes were replaced with the mean of the surrounding volumes. Additional preprocessing steps included: 8) spatial smoothing (FWHM) of 6 mm [3dBlurInMask, AFNI (73)]; 9) band-pass filtering between 0.01 and 0.08 Hz [3dFourier, AFNI (73)]; 10) linear and quadratic detrending [3dDetrend, AFNI (73)]; 11) regressing out nine nuisance regressors [all nuisance regressors were band-pass filtered with the same filter as in step 9: out of these, 6 were motion related (3 translations, 3 rotations) and 3 were anatomically related (not smoothed). Specifically, the anatomical nuisance regressors were: a) ventricles [Freesurfer (76), eroded in 2 mm space], b) draining veins (DVs) [FSL’s CSF minus Freesurfer’s Ventricles, eroded in 1 mm space (74, 76)], and c) local white matter (WM) [FSL’s WM minus Freesurfer’s subcortical gray matter structures, eroded in 2 mm space (74, 76)]. Regarding local WM regression, AFNI’s 3dLocalstat (73) was used to calculate the mean local WM time-series for each voxel, using a 25 mm radius sphere centered on each voxel (71)].

Values related to head motion (i.e., frame-wise displacement; FD) were significantly different between DMT and placebo (*P* = 0.003). While we employed significant motion correction methods, our findings could still be influenced by motion. To further validate the robustness of these findings, additional analyses were carried out using a subsample of eight participants showing no relationship between motion and connectivity using the same threshold for exclusion (FD = 0.4). This first subsample was identified by recursively removing participants until the correlation between Euclidian node distance and motion vs functional connectivity was no longer significant for the DMT condition (*SI Appendix*, Fig. S10). A second subsample was identified by using a stringent method of exclusion from analysis of FD = 0.2 (*SI Appendix*, Fig. S4), which resulted in only 3 participants. The results were broadly replicated in both analyses (*SI Appendix*, Figs. S2 and S3).

### EEG Preprocessing.

Gradient artifacts (GA) caused by the fMRI were removed using an average artifact template subtraction (AAS) algorithm which is part of the BrainVision Analyser software. The algorithm computes a representative template artifact based on a sliding average of 21 TR windows which is then subtracted from each TR window, thereby removing most of the MR-related noise (77). Following gradient artifact correction, the data were downsampled to 250 Hz and ballistocardiogram (BCG) artifacts were reduced by placing heartbeat markers corresponding to the R-peak determined on a representative template of the signal corresponding to the ECG channels (low-pass filtered at 15 Hz). An AAS algorithm was used to correct for the pulse artifact by producing a template resulting from averaging multiple cardiac cycles using a sliding-window approach, generating a different template for each sliding window, which is subtracted from that period (78). The following preprocessing steps were performed using the Fieldtrip software (79): The data were demeaned, band-pass filtered at 1 to 45 Hz, and epoched in separate 2-s trials. The data were then visually inspected and trials containing artifacts associated with jaw clenches and gross artifacts were removed. Independent component analyses were subsequently performed to remove residual BCG and GA artifacts as well as eye movements. If remaining gross artifacts were observed, the corresponding segments of the data were removed and ICA was ran again for improved results. Validation of preprocessing results was performed by comparing out-of-scanner EEG profile in the time and frequency domain, as well as in-scanner data. Two out of the 20 subjects were removed due to excessive data artifacts. Furthermore, as alpha analyses were performed on a fixed 8 to 13 Hz window, one subject exhibiting an alpha peak at 7.5 Hz was removed from group analyses as alpha analyses were performed on a fixed 8 to 13 Hz window. Participants where we had to discard (due to artifacts) 25% of the initial 8 min of data post DMT/placebo injection were removed from main EEG results presented in Fig. 4. No further participants reached the 25% threshold when considering the full 28-min scan. Compared with the fMRI data, a higher threshold of exclusion was used for the EEG, as this is convention, due to the higher temporal resolution and signal-to-noise ratio with EEG. EEG-fMRI analysis was conducted only for those participants who survived both the fMRI and EEG thresholds for exclusion (n = 12) (71).

## Data Analysis

### Within-Network Integrity (fMRI).

To analyze the integrity of the canonical resting-state networks (RSN), we employed the widely used seven-network parcellation by Yeo et al. (80) consisting in the visual network (VIS), somatomotor network (SM), dorsal attentional network (DAN), ventral attentional or salience network (SAL), limbic network (LIM), frontoparietal control network (FP), and the default-mode network (DMN).

Within-network integrity was determined for each of the 7 RSN for both the DMT and placebo scans by applying FSL’s dual regression analysis on the 7 relevant spatial components (networks). Dual regression first used the components as regressors applied to the four dimensional (4D) BOLD datasets of each subject, which resulted in a matrix of time-series for each IC. These time-series were then regressed into the same 4D scans to get a set of spatial maps specific to each subject [parameter estimate (PE) images]. For each subject, the mean PE across voxels was calculated for each of the DMT/placebo scans for each condition, within each of the 7 RSNs of interest, with the resulting PE representing the integrity at each RSN. Paired *t* tests were used to calculate the difference in integrity between conditions for each RSN (FDR corrected for multiple comparisons) (71).

### Between-Network Segregation (fMRI).

Network segregation was obtained using the same procedure as used in our previous work involving LSD and psilocybin (19, 21). A resting-state functional connectivity (RSFC) matrix was obtained representing connectivity between each of the 7 RSN of interest. The time-series from the first step of the dual regression for each pair of RSNs, were entered into a GLM, which resulted in PE values representing the strength of functional connectivity between pairs of RSNs. The GLM was done twice with each RSN as a dependent variable in one model and as an independent variable in the second model, which were then averaged, to generate two 7 × 7 matrices for DMT and placebo. The difference between DMT and placebo was established by *t* tests on each of the quadrants of the matrices (FDR corrected for multiple comparisons) (71).

### Pairwise Functional Connectivity (fMRI).

The degree to which connectivity between pairs of areas in the brain was altered during DMT administration was tested via a data-driven approach. First, the dimensionality of fMRI data was reduced by obtaining the average activity of voxels for each of the 100 areas associated with the Schaeffer atlas (37), plus 12 subcortical areas derived from the AAL atlas (81). Then, Pearson’s correlational analyses were performed between each pair of the 112 areas for both DMT and placebo administration, which resulted in a connectivity matrix for each condition consisting in 112*112 connections (or “edges”). Finally, paired *t* tests were performed for each of the edges, which resulted in the difference between DMT and placebo (FDR corrected for multiple comparisons) (71).

### Global Functional Connectivity (fMRI).

Global functional connectivity was obtained by taking the average of the normalized Fisher Z score of each Pearson’s correlation coefficient value from each area of the brain to all other areas of the brain. In order to reduce the number of statistical tests, we consider the 112 regions described above. Global connectivity at the network level was obtained by averaging the GFC values for all Schaeffer parcellations corresponding to each of the seven networks by Yeo et al. (71, 80).

### Dynamic Functional Connectivity (fMRI).

Dynamic connectivity matrices were calculated using a tapered sliding window approach (82). Sliding windows of 44 s (22 TRs) were convolved with a 6-s (3 TR) Gaussian kernel, with a 2-s step size obtained for the total of 28 min duration of the session (8 min baseline plus 20 min postinjection). In order to investigate dynamic changes of GFC (per area) and pairwise connectivity (per edge), we used real-time intensity ratings from DMT minus placebo sessions (also convolved with a 6-s Gaussian Filter). These rating changes were applied in linear mixed-effects models (one per brain region) using GFC (or pairwise FC) timecourse (DMT minus placebo) as the response variable and intensity timecourses as the predictor variable (with the intercept and slope as random variables, grouped per subject; FDR corrected for multiple comparisons). To corroborate our findings, we employed the same procedure using average DMT plasma levels corresponding to the 20 mg dose from our previous work (32) instead of subjective intensity ratings.

Additionally, we also explored significant moments in which GFC (network level) changes occurred during the timecourse of DMT effects by performing pairwise *t* test comparisons for each timepoint (i.e., each TR). This analysis was carried out globally and for each of the 7 resting-state networks used. Significant changes of average GFC (global and per network) between DMT and placebo were established via temporal cluster statistics, which were calculated over a null distribution obtained via permutations of the labels (DMT and placebo) of the time series for each subject (83).

### Overlap with Serotonin Receptor Density.

Comparisons between changes in dynamic GFC and density of serotonin receptors were made by taking the values for each of the Schaeffer atlas—plus 12 subcortical areas of the AAL atlas—derived from the linear effects model derived from the dynamic GFC analysis. This was carried out for the density maps of 5-HT2A receptors in each corresponding area (71), as obtained from Beliveat et al. (38).

### Spectral Analysis (EEG).

For spectral analysis, we performed Irregularly Resampled AutoSpectral Analysis (84) on the preprocessed signal to isolate the specific contribution of oscillatory components to spectral power (isolated from 1/f activity) following previous work (32, 84, 85), considering the confounders usually associated with 1/f activity on the EEG (84). The spectrum was then divided into the following frequency bands: delta (1 to 4 Hz), theta (4 to 8 Hz), alpha (8 to 13 Hz), beta (13 to 30 Hz), and gamma (30 to 45 Hz). For time-averaged spectral activity, we created blocks of eight minutes of data before and after injection for both DMT and placebo conditions. The comparison between both conditions was performed after subtracting the baseline (obtained before the injection) for each of them, in order to eliminate order effects and overall differences in power associated with preprocessing procedures. Spectral analyses were performed separately for each frequency band, and cluster-based permutation (7,500 permutations) testing of t-statistics was used to determine channels of significant differences between DMT and placebo (71).

### Signal Diversity (EEG).

Following our previous study involving DMT (32), as well as those performed with LSD, psilocybin, and ketamine (34), we performed signal diversity analysis using the Lempel–Ziv 1976 algorithm. The EEG signal at each single electrode was binarized using its mean for each 2-s epoch, and then the LZ76 algorithm was used to generate a dictionary of unique subsequences whose size quantifies the temporal diversity for the signal (denoted here as LZs). Fieldtrip cluster-based permutation (7,500 permutations) testing of t-statistics was used to determine channels showing significant differences between DMT and placebo. The average LZs across channels are denoted here as LZc. For all averaged analyses of EEG metrics, the 8-min baseline was subtracted from DMT and placebo conditions to account for potential differences in signal quality across visits (71).

### Dynamic Analysis of Spectral Activity and Signal Diversity.

We performed dynamic EEG analysis by calculating Pearson’s correlation coefficients between the average spectral power for each frequency band (and LZs) per epoch (DMT minus placebo), for each channel, and for each subject separately. The resulting values were then Fisher Z normalized and compared against a null distribution using cluster-based permutation (7,500 permutations) testing of t-statistics to determine channels of significant effects for each frequency band and LZs. Temporal-specific changes between DMT and placebo were established via temporal cluster statistics calculated over a null distribution obtained via permutations of the labels (DMT and placebo) of the time series (83). Permutations were done by inducing circular rotations on the data, hence keeping the autocorrelation and spectral properties of each time series untouched. At each permutation, the same algorithm was performed and the value of the largest cluster statistics (i.e., the sum of all T-scores within a cluster) was stored. These values were then gathered together to build a null distribution, against which the original cluster statistics were compared to evaluate their significance.

### Cortical Traveling Waves.

We quantified the amount and direction of traveling waves using the same analysis as in our previous study. First, we segmented the EEG signals in 1-s windows, using a sliding step of 500 ms. Next, we composed a 2D time-electrode map for each time window stacking five midline electrodes (i.e., Oz, POz, Pz, Cz, and FCz). We then performed a 2D fast Fourier transform on each map, from which we extracted the maximum value in the upper and lower quadrants, quantifying the power of forward and backward waves, respectively [see Alamia et al. (86) for more details]. To obtain a surrogate baseline, we performed the same analysis on each map after having randomized the electrodes’ order 100 times. This shuffling generates a surrogate 2D-FFT spectrum having the same temporal power but without spatial information (i.e., the waves’ directionality). Finally, we quantified the amount of forward and backward waves in decibels considering the log-ratio between the maximum values extracted from each quadrant in the 2D-FFT, and the average of the 100 repetitions in the surrogate spectra. Importantly, this value quantifies the amount of forward and backward waves as compared to the null hypothesis of having no cortical waves.

### EEG-fMRI Analysis.

We determined the relationship between EEG and fMRI measures by correlating the timecourse of EEG activity for each measure that was significant (delta, alpha, beta, and gamma power, and LZ) in the previous DMT vs. placebo comparison. We selected electrodes showing the highest statistical difference between both conditions and averaged their activity over time with its corresponding contralateral pair. Missing epochs (resulting from data cleaning) were omitted from the analysis, and epochs displaying activity over 5 SDs above the mean of the timecourse were interpolated. The data were band-pass filtered at 0.01 to 0.08 Hz and convolved with a canonical hemodynamic response function in order to match fMRI preprocessing. The resulting timecourses were then convolved with a 6-s Gaussian Filter to match the processing of fMRI dynamic functional connectivity matrices. Timecourses of GFC and pairwise functional connectivity were formed in the same way as described above (*Dynamic Functional Connectivity*). Data from 12 subjects survived preprocessing procedures for the full 28 min for both EEG and fMRI. Statistical analysis were performed using linear mixed-effects model using EEG timecourses as the response variable and GFC timecourse per brain region as the predictor variable (with the intercept and slope as random variables, grouped per subject; FDR corrected for multiple comparisons) (71). This use of linear mixed-effects models allowed us to exploit DMT-induced within-subject variability of brain metrics to find meaningful relationships between these (Fig. 5). Conversely, when employing an averaging approach of the time-courses (which disregards within-subject variability) before performing between-subject correlations, these relationships were less evident (*SI Appendix*, Figs. S8 and S9).

### Cortical Gradients.

Cortical gradients were computed using the BrainSpace toolbox [https://github.com/MICA-MNI/BrainSpace (87)] as implemented in MATLAB. Surfaces were first downsampled from fsaverage 5 space (20,484 vertices) to 10,000 vertices for computational efficiency. For each subject, a 10,000 × 10,000 connectivity matrix was then computed by calculating the pairwise Pearson’s correlation between all vertices. As has been done previously (27, 36), this matrix was z-transformed and thresholded row wise at 90% sparsity in order to retain only the strongest connections. Cosine similarity was then computed on the thresholded z-matrix in order to generate a similarity matrix which captures the similarity in whole-brain connectivity patterns between vertices. This similarity matrix is required as input to the diffusion map embedding algorithm we employed here. The use of cosine similarity as the similarity metric of choice is consistent with past work (36). Diffusion map embedding (36, 88), a nonlinear manifold learning technique from the family of graph Laplacians, was applied to similarity matrices in order to identify gradient components at the individual subject level. The technique estimates a low-dimensional set of embedding components (gradients) from a high-dimensional similarity matrix, where each embedding can intuitively be viewed as a dimension of FC pattern similarity covariance. In the embedding space, vertices that are strongly connected (as weighted by FC pattern similarity) by many connections or a few very strong connections are closer together, whereas vertices with little or no connections are farther apart. Euclidean distance between two points in the embedding space is equivalent to the diffusion distance between probability distributions centered at those points (hence the name of the algorithm), each of which is equivalent to “difference in gradient score” as referred to in the main text. The algorithm is controlled by a single parameter α, which controls the influence of density of sampling points on the manifold (α = 0, maximal influence; α = 1, no influence). Diffusion map embedding is specifically characterized by α = 0.5 (88), which allows the influence of both global and local relationships between data points in the estimation of the embedding space. Following past work (27, 89), to enable comparisons across subjects, Procrustes rotation was performed to align individual-subject embedding (gradient) components to an all-subjects group average embedding component template. This rotation ensures that gradient axes are matched across subjects. Group contrasts and behavioral associations were conducted using surface-based linear models, as implemented in the SurfStat toolbox (http://www.math.mcgill.ca/keith/surfstat) (90).

## Supplementary Material

Appendix 01 (PDF)

## Data Availability

fMRI, EEG and scripts for analysis data have been deposited in Github (https://github.com/timmer500/DMT_Imaging) (91).
