# Supplementary material for: Human brain effects of DMT assessed via EEG-fMRI
Source: Proc Natl Acad Sci U S A. 2023 Mar 20;120(13):e2218949120. doi: 10.1073/pnas.2218949120 (PMC10068756; doi:10.1073/pnas.2218949120)
Supplement: Supplementary file 1 — Appendix 01 (PDF) [file pnas.2218949120.sapp.pdf]

## Supplementary Information for
# Human brain effects of DMT assessed via EEG-fMRI

Christopher Timmermann*, Leor Roseman, Sharad Haridas, Fernando Rosas, Lisa Luan, Hannes Kettner, Jonny Martell, David Erritzoe, Enzo Tagliazucchi, Carla Pallavicini, Manesh Girn, Andrea Alamia, Robert Leech, David Nutt, Robin Carhart-Harris

Christopher Timmermann
Email: c.timmermann-slater15@imperial.ac.uk

**This PDF file includes:**

Figures S1 to S10
Table S1

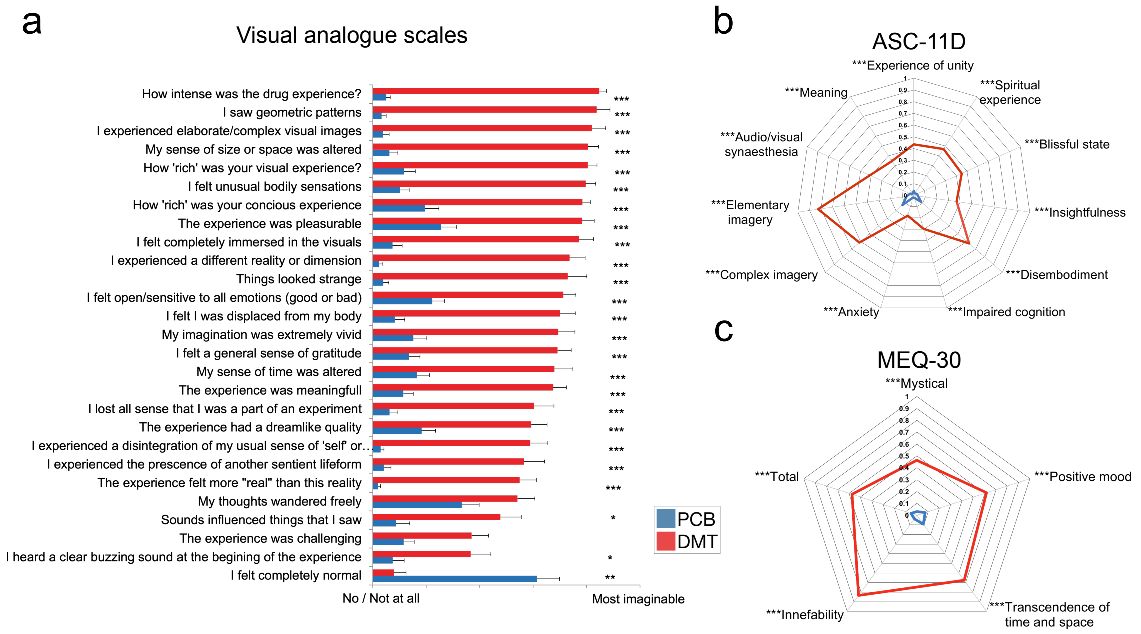


**Fig. S1. Subjective effects of DMT vs Placebo.** Comparative subjective effects of DMT and placebo (PCB) according to: (a) Visual analogue scales, (b) the 11 dimensions-Altered States of Consciousness Questionnaire (ASC-11D), and (c) the Mystical Experience Questionnaire (MEQ-30). (*p<0.05, **p<0.01, ***p<0.001; FDR-corrected).

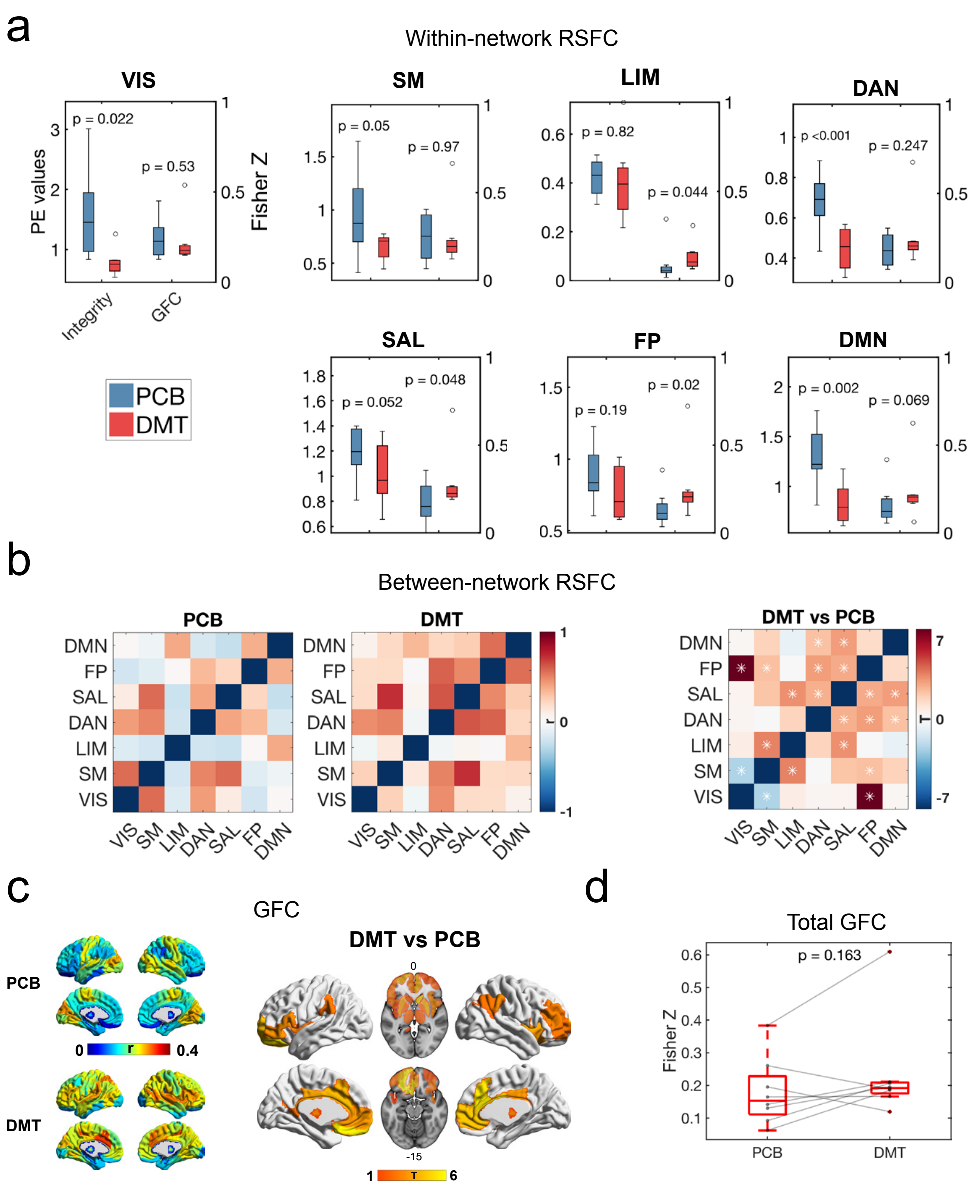


**Fig. S2. Effects of DMT on subsample without motion confounds on (n=8).** Effects of DMT on sRSFC on a subsample of subjects with reduced effects of head-motion (see Fig. S10) on (a) within-network integrity, (b) between-network connectivity, (c) local GFC, and (d) whole-brain GFC (non-corrected for multiple comparisons at p<0.05, due to small sample size). All findings correlating with head motion in the complete sample, where corroborated in this analysis and therefore the effect of motion may be ruled out. (sRSFC = static resting-state functional connectivity; GFC = global functional connectivity; networks; VIS = visual; SM = somatomotor; DAN = dorsal attentional; SAL = ventral attentional/salience; LIM = limbic; FP = frontoparietal; DMN = default-mode).

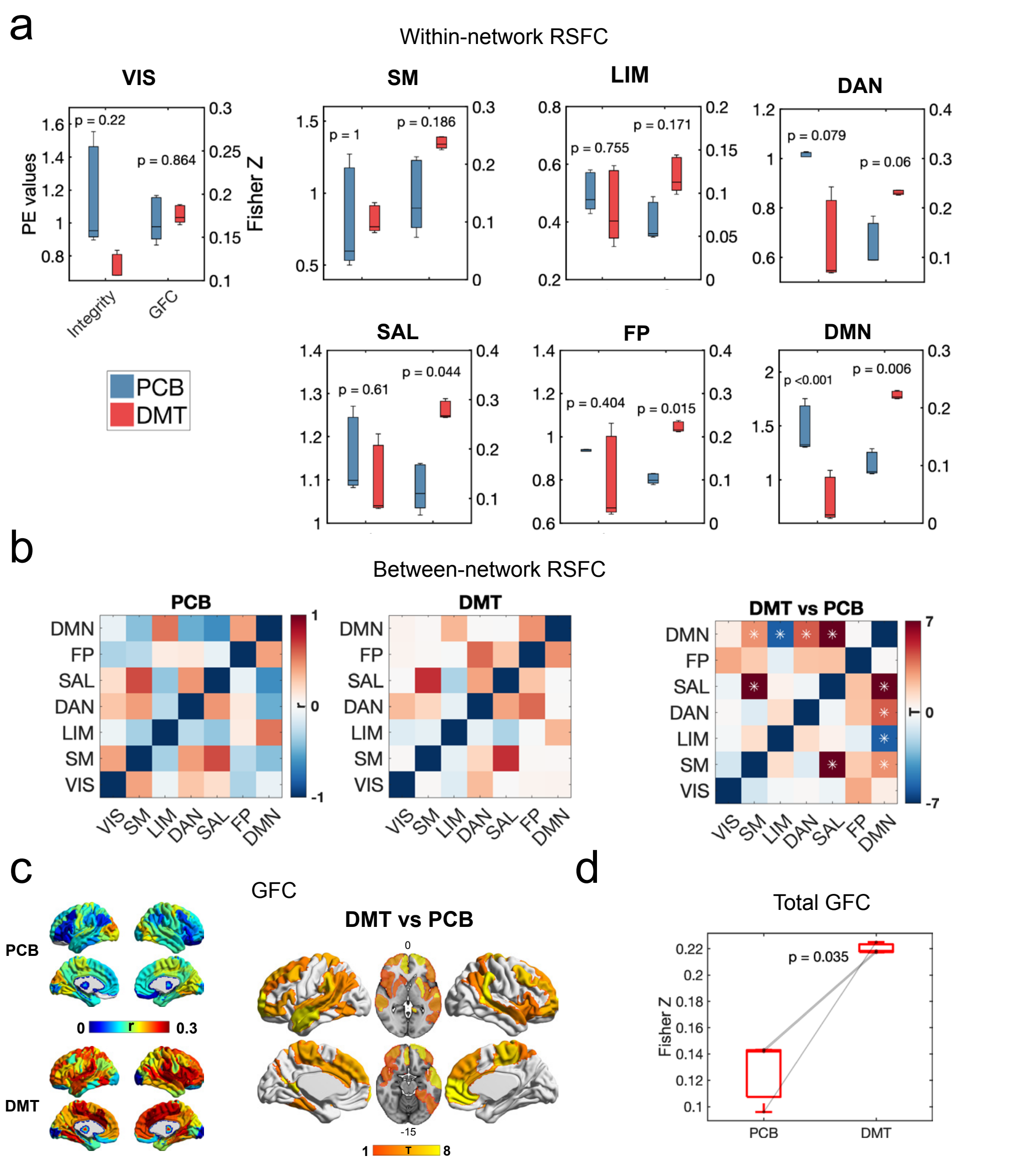


**Fig. S3. Effects of DMT on a subsample of participants (n = 3) showing minimal motion using a stringent threshold (FD = 0.2).** Effects of DMT on sRSFC in a subsample of subjects passing a stringent head-motion threshold of FD = 0.2 (see Fig. S4) for (a) within-network integrity, (b) between-network connectivity, (c) local GFC, and (d) whole-brain GFC (non-corrected for multiple comparisons and at $p<0.1$ due to small sample size). All comparisons between DMT and placebo are consistent with results reported in the main manuscript either in significance, at trend-level or in directionality. (sRSFC = static resting-state functional connectivity; GFC = global functional connectivity; networks; VIS = visual; SM = somatomotor; DAN = dorsal attentional; SAL = ventral attentional/salience; LIM = limbic; FP = frontoparietal; DMN = default-mode).

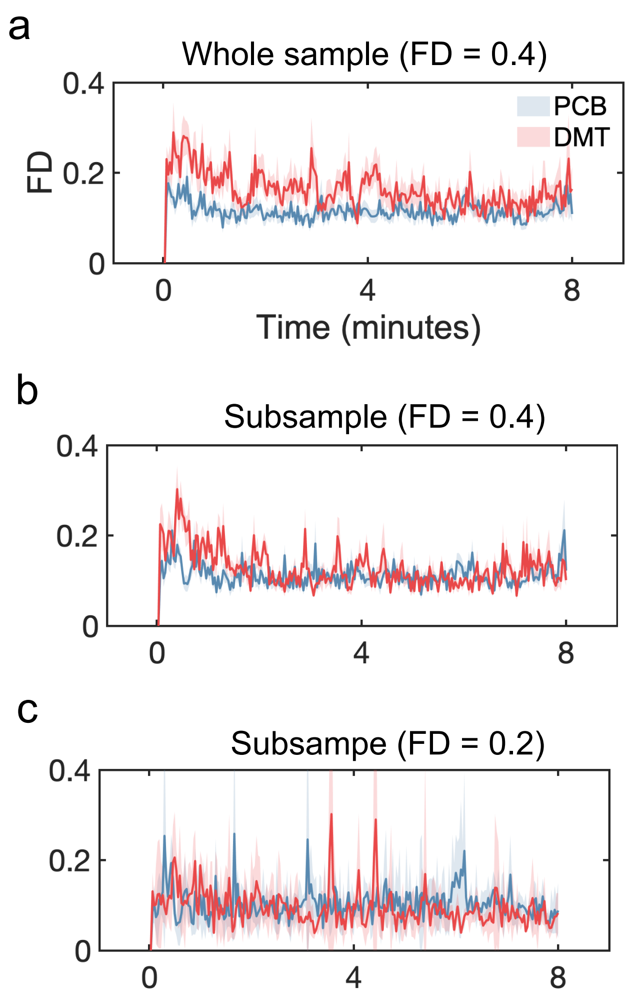


**Fig. S4.** Framewise displacement (FD) during the 8-minute period following DMT/Placebo. FD was significantly higher during DMT vs placebo in the initial 8 minutes of administration for the (a) whole sample ($p = 0.003$), but not for both (b) a subsample of individuals with no correlation between motion and connectivity ($p = 0.40$), and (c) a subsample of individuals surviving a stringent threshold on motion of FD = 0.2 ($p = 0.62$). Main analyses performed in this period, using both subsamples (Fig. S2-3) without motion confounds confirm the main effects seen for the whole sample (Fig. 1).

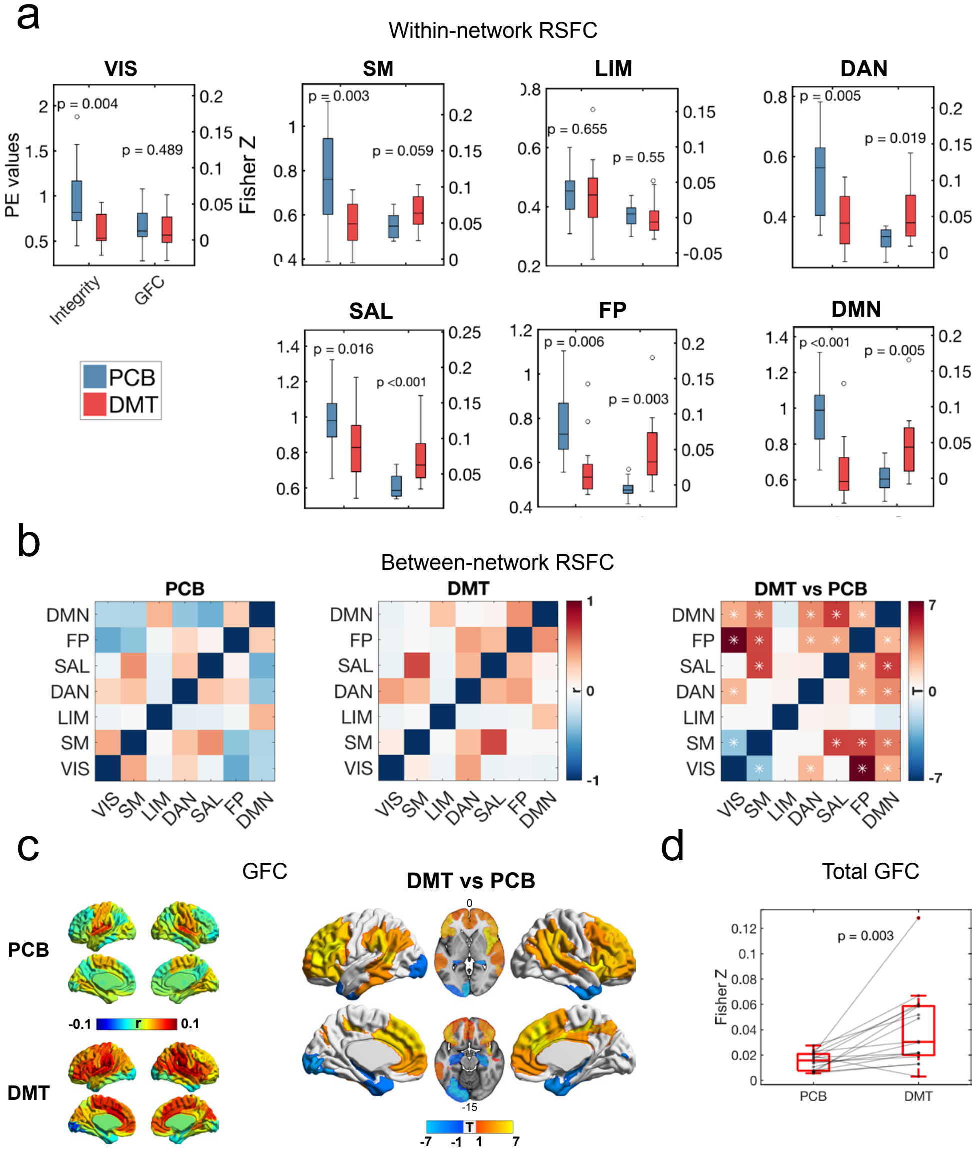

**Fig. S5. Effects of DMT using global signal regression (GSR).** (a) Analysis of within-network sRSFC or 'integrity' (parameter estimates and Fisher Z values) for DMT versus placebo shows significant reductions in integrity for 6 of 7 networks, and increases in GFC in 4 of 7 networks (FDR-correction, P < 0.05). (b) Decreased between-network segregation was especially pronounced between the FP/DMN/SAL or 'transmodal association pole' ('TOP') networks and other networks (*p<0.05, FDR-corrected) and increases in between-network segregation was found between SM and VIS networks. (c) Increases in GFC were especially pronounced for regions associated with the TOP of the human brain's principal gradient (p<0.05, FDR-corrected). (d) Total GFC (i.e., whole-brain) was found to be increased for DMT compared to placebo. (sRSFC = static resting-state functional connectivity; GFC = global functional connectivity; networks; VIS = visual; SM = somatomotor; DAN = dorsal attentional; SAL = ventral attentional/salience; LIM = limbic; FP = frontoparietal; DMN = default-mode; TOP = transmodal association pole).

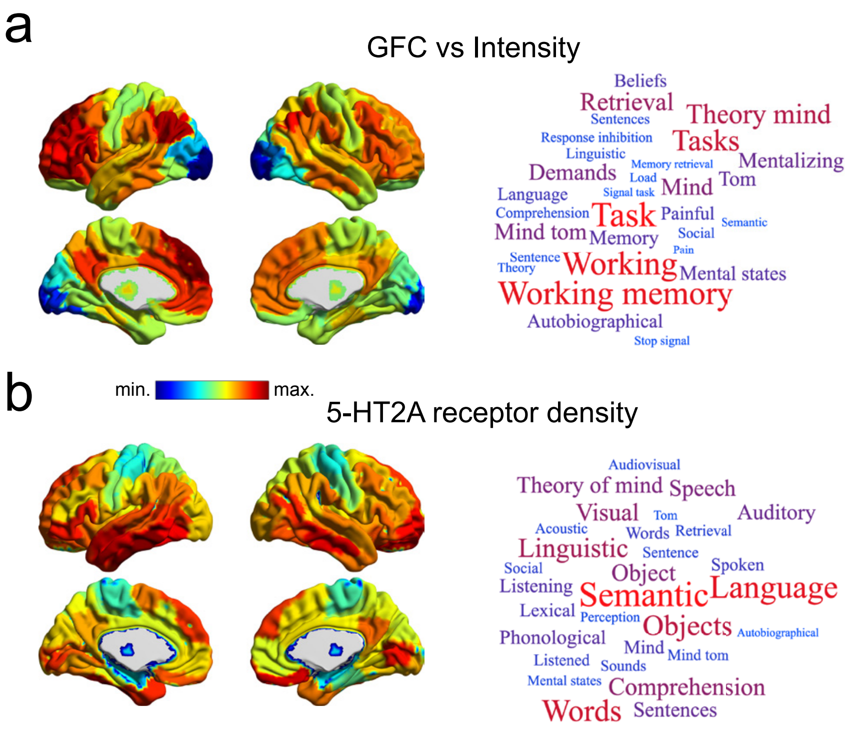


**Fig. S6. Association between DMT-induced GFC / 5-HT2A receptor density maps and brain function via Neurosynth.** (a) Map displaying dynamic (intensity-related) changes in GFC for DMT vs placebo (left) and word cloud displaying the first 30 non-anatomical terms obtained from Neurosynth associated with dynamic GFC results (right). (b) PET binding potential map for the 5-HT2A receptor (left) and the first 30 non-anatomical terms obtained from Neurosynth associated with the 5-HT2A receptor density map. GFC = global functional connectivity.

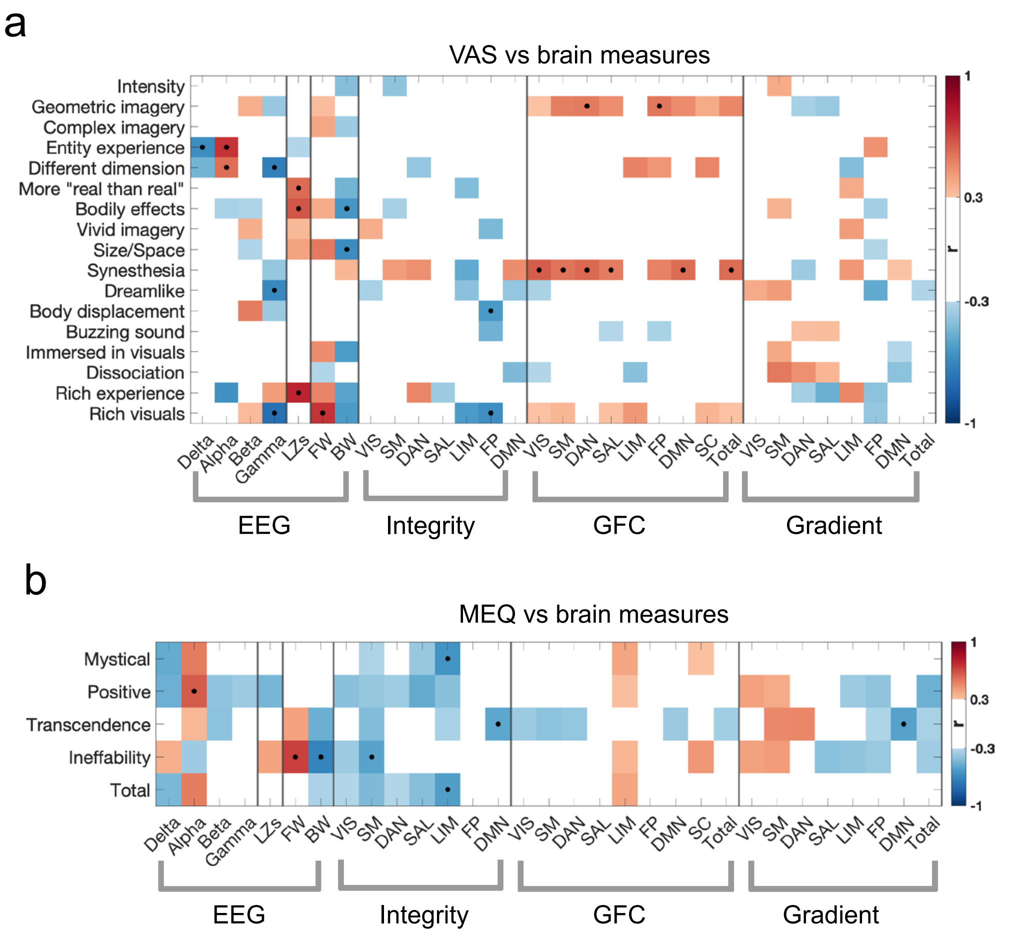


**Fig. S7. Exploratory correlations between DMT and subjective effects**. Exploratory correlations between DMT-induced changes in brain imaging metrics and subjective effects controlling for head motion (FD) by using partial correlations. (•p<0.05, not corrected for multiple comparisons). VAS = visual analogue scale. MEQ = mystical type experience questionnaire. VAS items and MEQ sub-factors are listed on the vertical axis of (a) and (b). Only moderate and strong correlations (i.e., r values of >0.3) are shown (red and blue blocks).

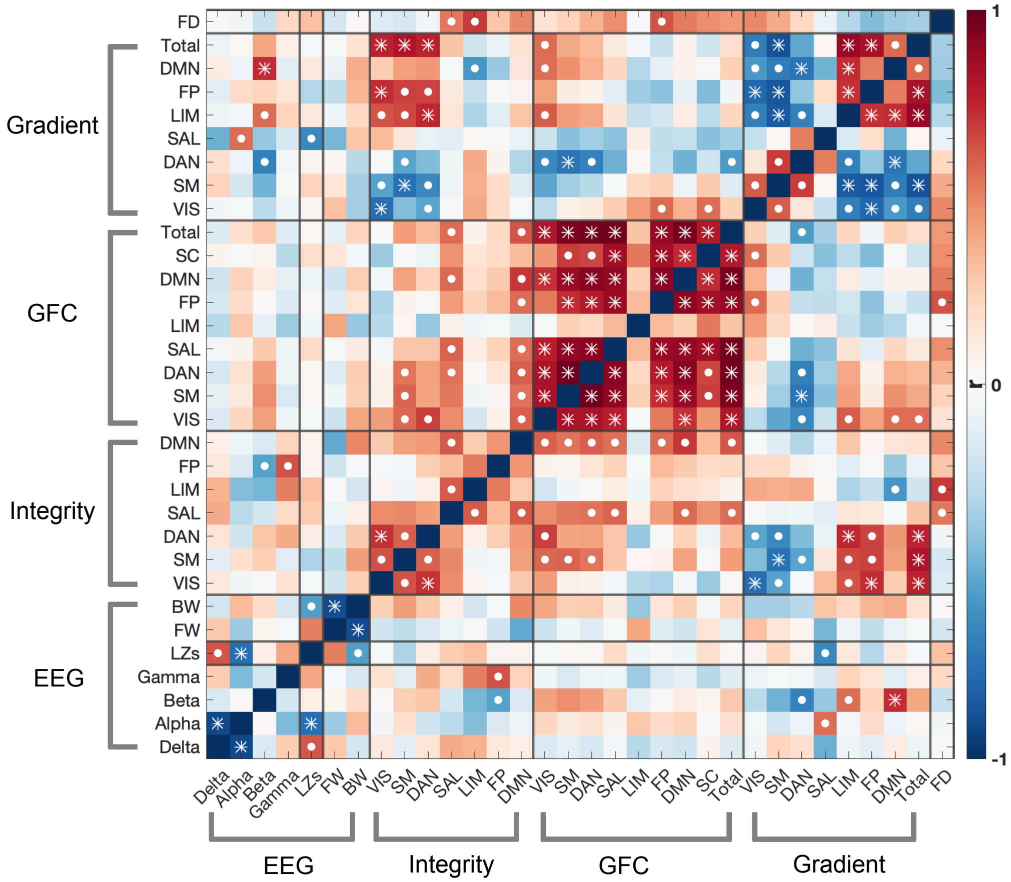


**Fig. S8. Correlations across measures and head motion (FD) between participants.** The correlation matrix displays associations between different fMRI measures, as well as between the fMRI measures and motion - determined via average FD before scrubbing (FD = top row, and farthest right column). All measures are computed as averages over the initial 8 minutes following the injection of DMT (as displayed in Fig. 1, 3, 4a, 4b), corresponding to peak effects (see Fig. 5 for an alternative approach which does not average within subjects but exploits within-subject dynamics for correlations). EEG metrics were taken as the averages across electrodes. •p<0.05, non-corrected; *p<0.05, FDR-corrected.

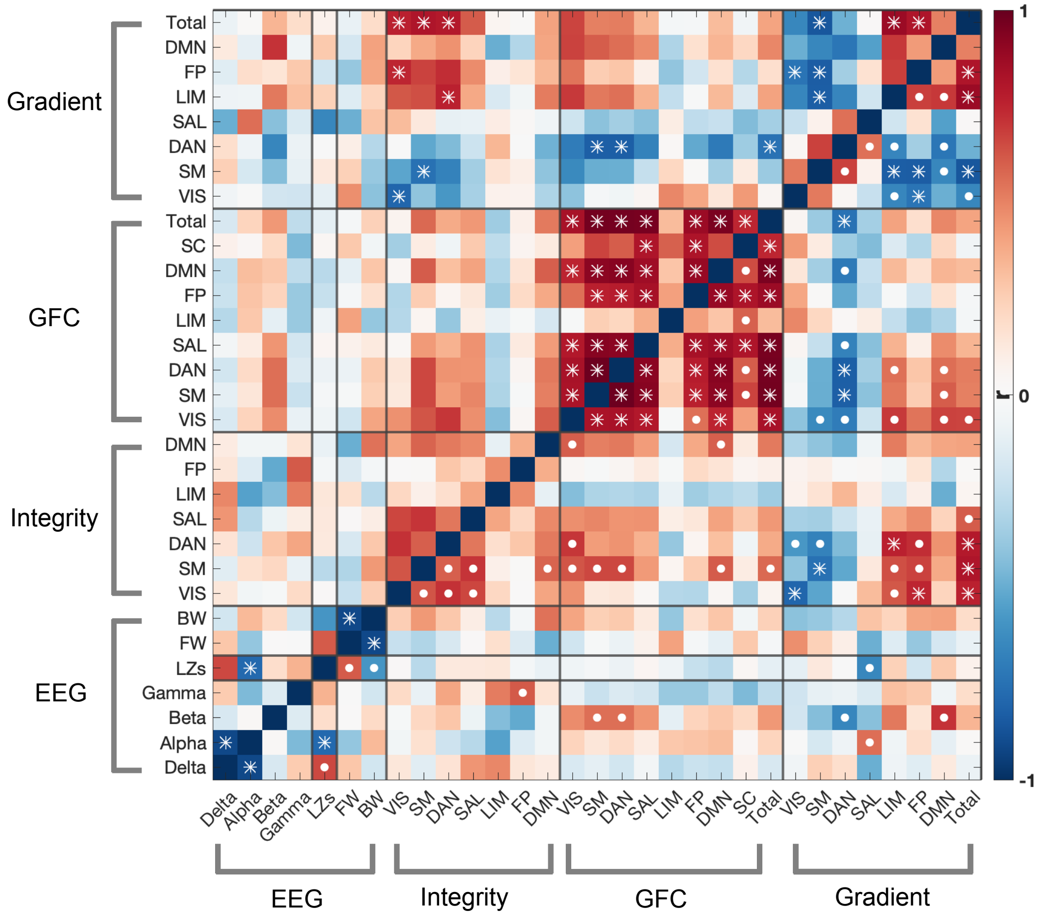


**Fig. S9. Correlations between neuroimaging measures across participants.** The correlation matrix displays associations between different fMRI and EEG measures controlled by head motion (FD) by using partial correlations. All measures were determined as averages during the initial 8 minutes following the injection of DMT (as displayed in Fig. 1, 3, 4a, 4b). EEG metrics were taken as the averages across electrodes. (•p<0.05, non-corrected; *p<0.05, FDR-corrected).

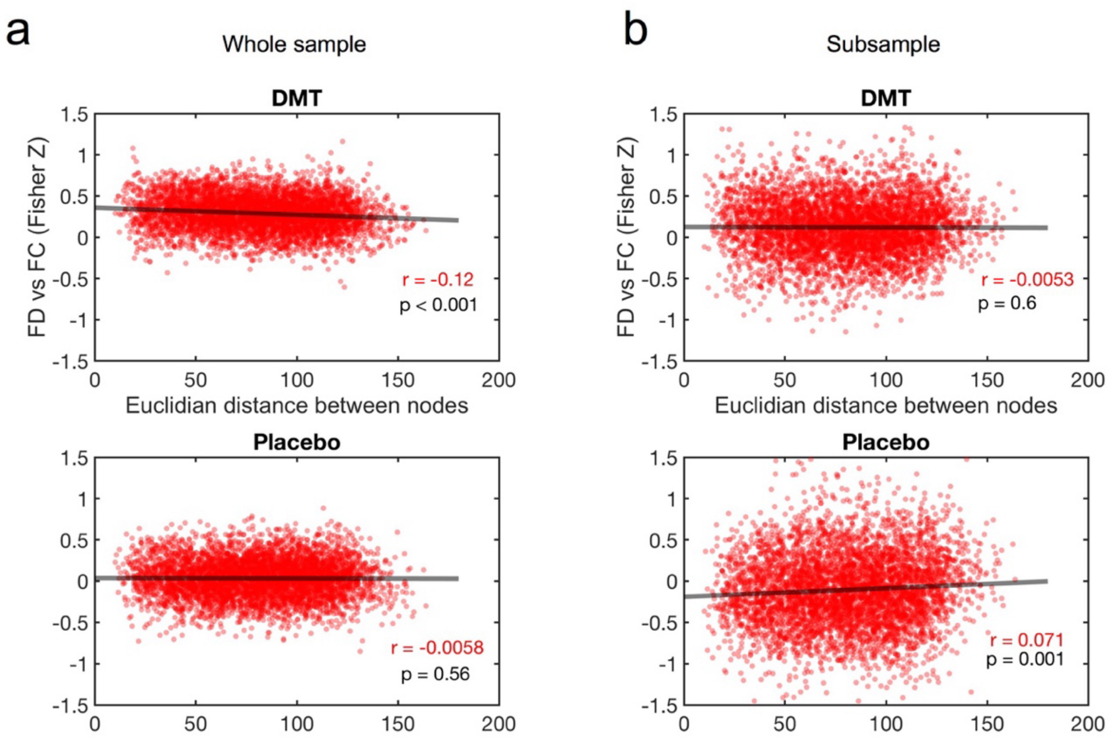


**Fig. S10. Relationship between node euclidian distance and fMRI-measured motion and functional connectivity correlation.** (a) scatter plots displaying the relationship between euclidian distance between brain nodes and motion vs functional connectivity (fisher Z converted Pearson correlation values) for the final sample used in analysis. (b) A subsample of 8 participants was selected by recursively removing subjects with higher motion from the sample until the correlation in DMT was no longer significant.

| | How difficult was it to stay awake? | | How tired were you? | | Percentage of the time spent asleep? | | Percentage of the time spent consciously trying to stay awake? | |
|---|---|---|---|---|---|---|---|---|
| **Measure** | **r** | **p** | **r** | **p** | **r** | **p** | **r** | **p** |
| Delta | -0.26 | 0.34 | -0.27 | 0.33 | -0.02 | 0.95 | -0.40 | 0.14 |
| Alpha | 0.24 | 0.38 | 0.35 | 0.20 | 0.01 | 0.97 | 0.43 | 0.11 |
| Gamma | -0.41 | 0.13 | **-0.54** | **0.04** | -0.51 | 0.05 | -0.15 | 0.61 |
| LZc | -0.46 | 0.08 | **-0.57** | **0.03** | -0.34 | 0.22 | -0.42 | 0.12 |
| FW | -0.16 | 0.58 | -0.36 | 0.19 | -0.07 | 0.80 | **-0.53** | **0.04** |
| BW | 0.17 | 0.55 | 0.34 | 0.22 | 0.17 | 0.53 | 0.43 | 0.11 |
| VIS Int | 0.33 | 0.22 | 0.14 | 0.62 | **0.62** | **0.01** | 0.03 | 0.91 |
| SM Int | 0.36 | 0.19 | 0.04 | 0.89 | **0.54** | **0.04** | 0.15 | 0.59 |
| DAN Int | 0.03 | 0.93 | -0.02 | 0.95 | 0.04 | 0.88 | -0.11 | 0.71 |
| SAL Int | 0.38 | 0.16 | 0.22 | 0.43 | 0.33 | 0.23 | 0.19 | 0.49 |
| FP Int | 0.01 | 0.96 | 0.10 | 0.72 | 0.13 | 0.64 | -0.25 | 0.37 |
| DMN Int | -0.06 | 0.83 | 0.08 | 0.77 | -0.28 | 0.31 | 0.03 | 0.92 |
| VIS GFC | 0.46 | 0.09 | 0.15 | 0.59 | 0.37 | 0.17 | 0.41 | 0.13 |
| SM GFC | 0.49 | 0.06 | 0.11 | 0.70 | 0.48 | 0.07 | 0.44 | 0.10 |
| DAN GFC | 0.48 | 0.07 | 0.13 | 0.64 | 0.45 | 0.09 | 0.49 | 0.07 |
| SAL GFC | 0.37 | 0.18 | 0.03 | 0.91 | 0.40 | 0.14 | 0.31 | 0.26 |
| LIM GFC | 0.32 | 0.25 | 0.18 | 0.53 | 0.46 | 0.08 | -0.01 | 0.96 |
| FP GFC | 0.32 | 0.24 | 0.10 | 0.72 | 0.41 | 0.12 | 0.29 | 0.29 |
| DMN GFC | -0.19 | 0.50 | -0.01 | 0.96 | -0.21 | 0.44 | -0.34 | 0.21 |
| Gradient | 0.16 | 0.57 | -0.18 | 0.51 | 0.43 | 0.11 | 0.09 | 0.76 |

**Table S1. Association between VAS of drowsiness and imaging metrics.** Questions regarding drowsiness were responded after DMT and placebo scanning sessions. Pearson correlation analyses were performed between DMT minus placebo drowsiness and imaging metrics showing the main outcomes reported in this paper. No contrasts survive FDR multiple comparison correction. If correction is neglected, 5 out of 100 comparisons reached significance (in bold), but due to the breadth of the multiple testing, these are susceptible to being false positives. VAS = visual analogue scale. Int = Integrity. GFC = global functional connectivity.
